# Novel *PUF60* variant suggesting an interaction between Verheij and Cornelia de Lange syndrome: phenotype description and review of the literature

**DOI:** 10.1038/s41431-023-01527-1

**Published:** 2024-01-25

**Authors:** Amarens Hoogenboom, Farah A. Falix, Liselot van der Laan, Jennifer Kerkhof, Mariëlle Alders, Bekim Sadikovic, Mieke M. van Haelst

**Affiliations:** 1 Medical University of Groningen (UMCG), Groningen, the Netherlands; 2 Department of pediatrics, Curaçao Medical Center (CMC), Willemstad, Curaçao; 3 Department of Human Genetics, Amsterdam UMC, Amsterdam, the Netherlands; 4 Amsterdam Reproduction & Development, Amsterdam University Medical Centers (AUMC), University of Amsterdam, Amsterdam, The Netherlands; 5 Verspeeten Clinical Genome Centre, London Health Sciences Centre, London, ON Canada; 6 Department of Pathology and Laboratory Medicine, Western University, London, ON Canada; 7 Emma Center for Personalized Medicine, Amsterdam UMC, Amsterdam, The Netherlands

**Keywords:** DNA methylation, Clinical epigenetics

## Abstract

Verheij syndrome [VRJS; OMIM 615583] is a rare autosomal dominant neurodevelopmental disorder characterized by distinct clinical features, including growth retardation, intellectual disability, cardiac, and renal anomalies. VRJS is caused by deletions of chromosome 8q24.3 or pathogenic variants in the *PUF60* gene. Recently, pathogenic *PUF60* variants have been reported in some individuals with VRJS, contributing to the variability in the clinical presentation and severity of the condition. *PUF60* encodes a protein involved in regulating gene expression and cellular growth. In this report, we describe a new case of VRJS with developmental delay, cardiac-, and renal abnormalities, caused by a heterozygous pathogenic *PUF60* variant. Surprisingly, DNA methylation analysis revealed a pattern resembling the Cornelia de Lange syndrome (CdLS) episignature, suggesting a potential connection between *PUF60* and CdLS-related genes. This case report further delineates the clinical and molecular spectrum of VRJS and supports further research to validate the interaction between VRJS and CdLS.

## Introduction

Verheij syndrome (VRJS (OMIM #615583)) is characterized by growth retardation, intellectual disability, irido-retinal or chorioretinal coloboma, dysmorphic facial features, cervical spine segmentation defects, cardiac, and renal abnormalities [1]. VRJS is caused by deletions within chromosome 8q24.3 typically spanning from 78 kb to 1 Mb. Interestingly, within this observed range no identical breakpoints have been identified. Within this context, a common minimal deletion was identified [2], encompassing three genes: *SCRIB*, *NRBP2* and *PUF60* [3]. Subsequently, the identification of loss of function variants in *PUF60* in patients with similar phenotypes confirmed *PUF60* to be responsible for VRJS S [3]. To date, 56 patients with VRJS and a pathogenic *PUF60* variant have been described [4].

*PUF60 (*OMIM #604819), also known as poly-U-binding factor 60 kDa, encodes a splicing factor involved in 3′ splice-site recognition and plays a role in transcription regulation. *PUF60* interacts with the U2 auxiliary factor (U2AF) in RNA binding and splicing activation and is widely expressed during embryonic development [5].

Given its role in epigenetic regulation, we hypothesized that *PUF60* abnormalities may be associated with a distinct DNA methylation pattern. Apart from clinical application of DNA methylation signatures to reevaluating variants of unknown significance (VUS), they can also serve as a diagnostic resource for individuals with suspected genetic disorders and unconfirmed molecular diagnoses [6]. Beyond their diagnostic roles, DNA methylation analyses can offer valuable insights into the molecular mechanisms underlying genetic disorders [7]. To date, more than 56 episignatures associated with 65 genetic syndromes have been published and consequently implemented in clinical diagnostics through EpiSign^TM^ classifier technology [8].

In this study, we describe a new patient with VRJS caused by a novel *PUF60* variant and we (1) expand the phenotypic spectrum of VRJS and review the literature, and (2) since no episignature has been associated with *PUF60* as yet, we aim to explore whether our patient exhibits a DNA methylation pattern that overlaps with any of the 65 genetic syndromes.

## Methods

Array-CGH microarray involved the use of peripheral blood and 180k microarray slides from Agilent Technologies (Santa Clara, CA), following the manufacturer’s protocols. Scanned images were processed using Agilent Genomic Workbench version 6.5 and Cartagenia software (Agilent Technologies). Interpretation of Copy Number Variation (CNV) data followed the lab’s guidelines.

Karyotyping was performed with GTG-banding (Giemsa-Trypsin-Giemsa banding) and analyses were performed on Cytovision version 7 based on guidelines of the Amsterdam UMC.

Whole exome sequencing (WES), with a filter for intellectual deficit (ID) genes was performed using KAPA HyperExome capture arrays (Roche) with next generation sequencing (NGS) on an Illumina sequencing platform. Sequence reads were mapped against the human reference genome (NCBI build 37/hg19) using BWA 0.7.10 (bio-bwa.sourceforge.net/), and variants were annotated using GATK Version = 3.3-0-g37228af (www.broadinstitute.org/gatk/). Filtering of variants was done using Alissa Interpret NGS (Agilent). Variants with a minor allele frequency >0.02% for dominant disorders or >1% for recessive disorders in GnomAD v2.1.1, as well as variants outside exon +/- 6 nucleotides, which are and not listed as (likely) pathogenic in ClinVar or HGMD databases were excluded from analysis. All other variants were evaluated.

The DNA methylation profile of our patient was examined through EpiSign^TM^ testing [8]. Since no episignature has been associated with *PUF60* as yet, we aimed to explore whether our patient exhibited a DNA methylation pattern that overlapped with any of the 65 genetic syndromes in parallel with the WES ID [8].

## Results

### Case report

The index patient is a 4-year-old boy. He was born by cesarean section at 39 weeks and 3 days of gestation with Apgar scores of 7 and 9 at one and five minutes after birth, respectively. The pregnancy was achieved through in vitro fertilization (using both donated oocytes and sperm) and was complicated by gestational diabetes. Prenatal ultrasound showed mild hydronephrosis of the right kidney without signs of vesicoureteral reflux, which normalized in the third trimester. At birth, weight was 3550 g (at the 66th percentile), length: 47.5 cm (at the 10th percentile), and head circumference: 37 cm (at the 98th percentile). No dysmorphic features were observed.

During the neonatal period, a critical coarctation of the aorta, atrial septal defect, multiple ventricular septal defects, and a bicuspid aortic valve were detected, for which he underwent surgical correction with a successful post-operative outcome. Antihypertensive drugs were prescribed for a few months, after which cardiac function normalized. Due to the antenatal right-sided hydronephrosis, renal ultrasound was performed showing mild bilateral dilatation of the ureter-pelvis junction, which later spontaneously resolved. A dimercaptosuccinic acid (DMSA) scan of the kidneys revealed asymmetrical differential renal function distribution, with 35% on the right side and 65% on the left side, suggestive of right renal dysplasia. At follow-up, both the right and left kidneys showed normal growth without hydronephrosis.

Further work-up for other congenital anomalies in the neonatal period revealed hemivertebra of L4 without scoliosis. He experienced recurrent episodes of laryngitis subglottica. A laryngoscopy at the age of two years showed, apart from an enlarged adenoid, no laryngomalacia or other abnormalities. He underwent orchidopexy at the age of 3 years for cryptorchidism. Brain MRI performed at the age of 9 months revealed slightly increased peripheral cerebrospinal fluid space with otherwise normal cerebral anatomy.

At the age of two years, he was mildly delayed in development, especially in speech and motor skills, both of which improved with physiotherapy. He had frequent loose stools, occurring multiple times a day, and celiac and biochemical screening revealed no abnormalities. His growth and weight gain were normal during these years, without signs of malabsorption. His loose stools improved after the age of 4 years. He had leg length discrepancy of 5 mm due to the hemivertebra. Clinical dysmorphic evaluation showed a relatively short neck, prominent eyebrows and eyelashes, a thin upper lip, and an upturned nose. No external eye abnormalities were observed.

### Molecular analyses and DNA methylation profiling

**Fig. 1:**
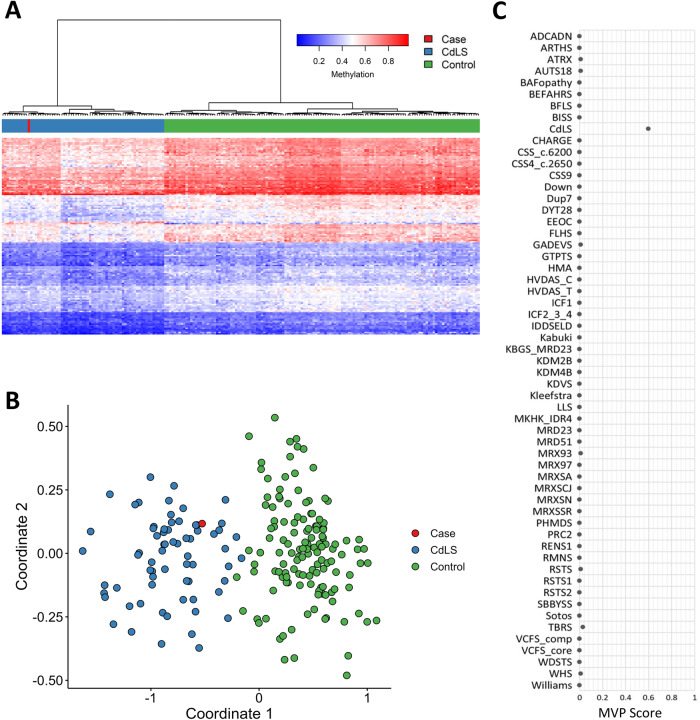
Confirmed episignature for our case. **A** Hierarchical clustering and **B** multidimensional scaling plots demonstrate the clustering of our case (shown in red) with the CdLS cases (marked in blue), which is distinct from controls (individuals without CdLS or VRJS; shown in green) **c** The methylation variant pathogenicity (MVP) score range from 0 to 1. Scores. Scores approaching 1 indicate a high likelihood of a methylation pattern characteristic of the target syndrome (CdLS), while scores close to 0 indicating a methylation profile similar to controls. Our case is showing an high MVP score, suggesting the presence of the CdLS episignature.

Genetic analysis at birth, including microarray and karyotyping, did not reveal any abnormalities. At the age of 4 years, whole exome sequencing (WES) with a filter for intellectual deficit (ID) genes revealed a heterozygous deletion of a single nucleotide, guanine (G) (c.688del), in exon 8 of the in *PUF60* gene (NM_078480.3). This results in a frameshift starting from position 230 and a stop codon after 58 positions in the new reading frame, p.(Val230Trpfs*58), which is predicted to undergo nonsense-mediated decay [9]. This variant was classified as likely pathogenic based on ACMG classifiers PVS2 (truncating in a gene where loss of function is a known mechanism of disease) and PM2 (absent in GnomAD databases). [10] Parental samples were not available to determine de novo occurrence of the variant. Similar frameshift variants in the same exon, have been reported de novo in literature [11–13]. Surprisingly, DNA methylation analyses revealed a DNA methylation profile similar to the Cornelia de Lange syndromes 1–4 (CdLS) episignature, associated with variants in *NIPBL, SMC1A, SMC3*, and *RAD21* (Fig. 1). Reanalysis of WES data including intronic variants specific for these genes did not reveal any (possibly) pathogenic variants.

## Discussion

We present a new patient with VRJS caused by a novel *PUF60* variant. This case expands the list of clinical features associated with *PUF60* variants causing VRJS [4]. The current patient exhibited severe cardiac anomalies, including aortic coarctation, an atrial septal defect (ASD), multiple ventricular septal defects (VSDs), and a bicuspid aortic valve. These cardiac abnormalities have been reported in other cases of VRJS (Table 1) [3, 4, 11–17]. However, aortic coarctation has been observed in only two patients: one with a deletion of chromosome 8q24.3 encompassing *SCRIB*, *NRBP2*, and *PUF60*, and another with a de novo variant in the splice acceptor site of exon 8 of the *PUF60* gene (c.604-2 A > C) [2, 11]. This suggests a possible association between *PUF60* and aortic coarctation in VRJS.

**Table 1:** Comparison of the clinical features observed in our patient with those previously documented in individuals with a *PUF60* gene variant.

Clinical phenotypes	Present case	Previous reported cases with *PUF60* variants (*n* = 56)
Auditory	-	16/40 (40%)
Cardiac	+	31/52 (60%)
Coloboma	-	19/49 (38%)
Developmental delay	+	49/54 (90%)
Genital	+	5/25 (20%)
Stool difficulties	+	2/10 (20%)
Hand analomies	-	26/49 (53%)
Joint laxity/dislocation	-	17/43 (40%)
Laryngeal	+	2/2 (100%)
Ocular	-	29/48 (60%)
Oral/Palatal deformities	-	16/32 (50%)
Renal	+	11/47 (23%)
Vertebral	+	30/50 (60%)

Various renal disorders, such as renal agenesis, pelvic kidney, horseshoe kidney, hypoplastic kidney, and hydronephrosis, have been previously reported in VRJS [3, 4, 11, 13, 15, 17]. The renal phenotype of the current patient was milder than other reports and appears to have normalized during development.

Other reported clinical abnormalities such as retinal coloboma, hand deformities, joint laxity/dislocation, seizures, feeding difficulties, and auditory problems were not observed in our patient [4]. This discrepancy could possibly be attributed to the location of this new variant. It’s worth noting that more than half of the reported patients with a *PUF60* variant exhibit skeletal involvement, most commonly presenting as scoliosis (Table 1) [3, 4, 11, 13, 15]. Our patient has a hemivertebra of L4, with no signs of scoliosis as yet, it is however important to monitor for potential scoliosis development during follow-up clinical evaluation.

Available literature on the urogenital and endocrine aspects related to *PUF60* is limited. Two cases have been reported with cryptorchidism [4, 15]. In addition, Moccia et al. [16] described a patient with unspecified genital abnormalities. Furthermore, Grimes et al. [4] reported a patient with small genitalia and delayed puberty who was treated with testosterone. Our report strengthens the suggestion of an association between *PUF60* variants and urogenital defects. Consequently, follow-up evaluations of urogenital and endocrine aspects in patients with *PUF60* variants is indicated.

Our patient experienced recurrent subglottic laryngitis, requiring repeated corticosteroid therapy and occasional hospital admissions. Laryngoscopy did not reveal any signs of upper airway malacia, and his symptoms gradually improved over the years. Apart from one previously reported case of laryngotracheobronchomalacia, without further specification, no other laryngeal or upper airway abnormalities have been documented in patients with VRJS [3].

Developmental delays in communication, motor skills, and intellectual disability are frequently reported (90%) (Table 1). The current patient exhibited mild psychomotor delay and speech delay, which improved after physio- and speech therapy. While behavioral difficulties are often reported in patients with a *PUF60* variant [4] they were not reported in our patient. During clinical observations, we noted friendly, social, and cooperative behavior.

The dysmorphic features of our case (a relatively short neck, prominent eyebrows and eyelashes, a thin upper lip, and an upturned nose) overlap with previous reported cases [3, 4, 11, 13]. Although gastro intestinal problems were reported some cases, this was not present in our patient [3, 11, 13]. Grimes et al. [4] documented a patient with chronic diarrhea from the age of 4 months to 4 years old and another patient with milk protein intolerance. These findings correlate with the report of loose stools, which resolved at the age of 4 years in our patient.

The results of the EpiSign^TM^ test revealed the CdLS episignature which is associated with variants in; *NIPBL, SMC1A, SMC3* and *RAD21* (Fig. 1). CdLS is characterized by a range of physical and intellectual disabilities and these genes are involved in the regulation of cohesion, a protein complex essential for chromosome structure and function [18]. While *PUF60* is involved in various cellular processes, including pre-mRNA splicing and a developmental role, a connection on protein level between *PUF60* and the genes that belong to CdLS signature has not been reported before. However, previous reports have indicated that at the phenotype level, patients with *PUF60* variants exhibit overlapping features with CdLS. These include facial hypertrichosis and prominent eyebrows. As a result, some patients with these features underwent targeted sequencing for CdLS which yielded negative results, only to later discover a pathogenic *PUF60* variant [11]. Our patient also displays prominent eyebrows and eyelashes, a thin upper lip, and a slightly upturned nose, which are also features that are seen in patients with CdLS. Unfortunately no consent for publication of facial photographs was given.

In conclusion, this report describes a patient with a novel variant in the *PUF60* gene, resulting in clinical manifestations of VRJS. In contrast to the more severe cardiac abnormalities (Coarctation of the aorta and multiple VSDs), the renal-, vertebral-, and developmental abnormalities observed in our patient are relatively mild compared to previously reported cases. Additionally, our patient experienced mild upper airway problems, including frequent episodes of laryngitis subglottica, and recurrent loose stools during the first year of life, which gradually improved over time. The results of the EpiSign^TM^ test suggest a potential link between *PUF60* and the established CdLS genes. Based on the previously reported overlap of dysmorphic features between CdLS and VRJS and the overlapping CdLS episignature in our case, we suggest a potential connection between *PUF60* and the known CdLS genes (*NIPBL*, *SMC1A*, *SMC3*, and *RAD21*). Further research with a larger number of patients is needed to confirm and validate this finding.

## Data Availability

Data are available from the authors on reasonable request.
